# Correction: Möller et al. Melanogenesis Is Directly Affected by Metabolites of Melatonin in Human Melanoma Cells. *Int. J. Mol. Sci.* 2023, *24,* 14947

**DOI:** 10.3390/ijms26073010

**Published:** 2025-03-26

**Authors:** Jack K. S. Möller, Kinga Linowiecka, Maciej Gagat, Anna A. Brożyna, Marek Foksiński, Agnieszka Wolnicka-Glubisz, Elżbieta Pyza, Russel J. Reiter, Meri K. Tulic, Andrzej T. Slominski, Kerstin Steinbrink, Konrad Kleszczyński

**Affiliations:** 1 Department of Dermatology, University of Münster, Von-Esmarch-Str. 58, 48149 Münster, Germany; j_moel40@uni-muenster.de (J.K.S.M.); kerstin.steinbrink@ukmuenster.de (K.S.); 2 Department of Human Biology, Faculty of Biological and Veterinary Sciences, Nicolaus Copernicus University, Lwowska 1, 87-100 Toruń, Poland; klinowiecka@umk.pl (K.L.); anna.brozyna@umk.pl (A.A.B.); 3 Phillip Frost Department of Dermatology & Cutaneous Surgery, University of Miami Miller School of Medicine, Miami, FL 33125, USA; 4 Department of Histology and Embryology, Collegium Medicum in Bydgoszcz, Nicolaus Copernicus University in Toruń, 85-092 Bydgoszcz, Poland; mgagat@cm.umk.pl; 5 Department of Clinical Biochemistry, Faculty of Pharmacy, Collegium Medicum in Bydgoszcz, Nicolaus Copernicus University in Toruń, 85-092 Bydgoszcz, Poland; marekf@cm.umk.pl; 6 Department of Biophysics and Cancer Biology, Faculty of Biochemistry, Biophysics and Biotechnology, Jagiellonian University, Gronostajowa 7, 30-387 Krakow, Poland; a.wolnicka-glubisz@uj.edu.pl; 7 Department of Cell Biology and Imaging, Institute of Zoology and Biomedical Research, Jagiellonian University, Gronostajowa 9, 30-387 Kraków, Poland; elzbieta.pyza@uj.edu.pl; 8 Department of Cell Systems and Anatomy, UT Health, Long School of Medicine, San Antonio, TX 78229, USA; reiter@uthscsa.edu; 9 Team 12, INSERM U1065, Centre Méditerranéen de Médecine Moléculaire (C3M), Université Côte d’Azur, 06200 Nice, France; meri.tulic@unice.fr; 10 Department of Dermatology, Comprehensive Cancer Center, University of Alabama at Birmingham, Birmingham, AL 35294, USA; aslominski@uabmc.edu; 11 Pathology and Laboratory Medicine Service, VA Medical Center, Birmingham, AL 35294, USA

In the original publication [1], there was a mistake in Figure 5 and the legend. There was an overlap between panels in Figure 5. The authors state that the scientific conclusions are unaffected. This correction was approved by the Academic Editor. The original publication has also been updated. The corrected Figure 5 and legend appears below.

## Figures and Tables

**Figure 5:**
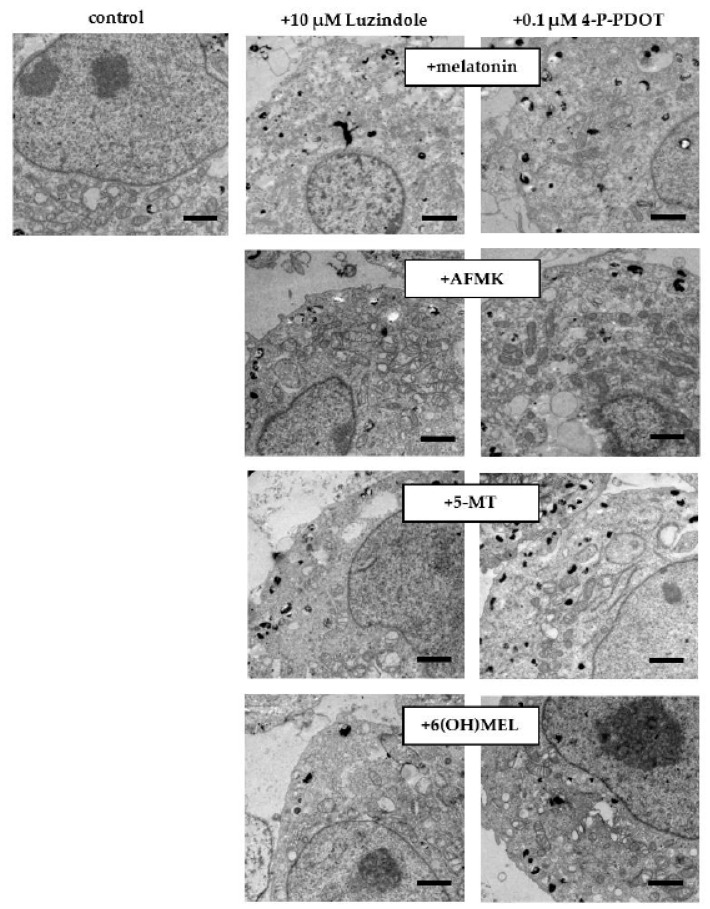
Melatonin and its metabolite-induced drop in melanin content in melanotic MNT-1 melanoma cells. After 72 h incubation with melatonin and its metabolites (10^–3^ M), transmission electron microscopy (TEM) images were obtained as described in *Materials and Methods,* where the effects of G-coupled membrane receptors (10 μM luzindole or 0.1 μM 4-P-PDOT) were assessed, and their presence did not affect the collapse of melanogenesis. Bars: 1 μm.
